# Experimental investigation of the responses of meadow buttercup (*Ranunculus acris* L.) to sodic salinity and its implications for habitat monitoring

**DOI:** 10.1038/s41598-023-42738-2

**Published:** 2023-09-20

**Authors:** Mateusz Wala, Jeremi Kołodziejek, Janusz Mazur, Jacek Patykowski

**Affiliations:** 1 Department of Geobotany and Plant Ecology, Faculty of Biology and Environmental Protection, University of Łódź, Banacha 12/16, 90-237 Łódź, Poland; 2 Laboratory of Computer and Analytical Techniques, Faculty of Biology and Environmental Protection, University of Lodz, Banacha 12/16, 90-237 Łódź, Poland; 3 Unaffiliated, Łódź, Poland

**Keywords:** Plant ecology, Plant stress responses, Ecophysiology, Grassland ecology, Invasive species

## Abstract

*Ranunculus acris* L. is a native species widely distributed throughout Europe and is invasive in nonnative areas, causing substantial economic losses in pasture productivity. The present study examined the effects of sodic salinity on the growth and functioning of this species. Salinity stresses the germination process and seedling growth, indicating that the studied species experience serious limitations at 60–90 mmol dm^−3^ NaCl and cannot establish in habitats where salinity is equal to or greater than 150 mmol dm^−3^ NaCl. *R. acris* is tuned to subsaline habitats characteristic of temperate meadows, as its growth and functioning were the best when the plants were treated with 30 mmol dm^−3^ NaCl. Increasing salinity (60 and 90 mmol dm^−3^ NaCl) hampered growth, leaf morphology and photosynthesis but not mineral nutrition, as Na accumulation seemed to be the most outlined effect of NaCl application. Changes in leaf morphological characteristics coordinated well with Na content in those organs, which indicates that leaf appearance can be easily catchable sign of progressing salinity. Ultimately, progressing salinity reduces the competitiveness of the studied species, shifting its strategy to ruderal behavior, but under subsaline conditions, the strategy of this species seems to be most balanced.

## Introduction

Meadow buttercup (syn. tall buttercup, giant buttercup), *Ranunculus acris* L. (Ranunculaceae), is a polycarpic perennial herb native to Europe and western Asia (Siberia^1^). It has also been introduced into North America, South Africa, Asia, and New Zealand^2^. *R. acris* is tolerant of a wide range of habitats, including natural, seminatural and strictly man-made habitats (e.g., meadows, pastures, wastelands, roadsides, railroad embankments and forest clearings^1^). Although it is believed to be a species with wide tolerance to edaphic conditions, drought can limit its occurrence^1^. Due to its relatively wide tolerance to environmental conditions, it is considered a serious weed within both native and nonnative ranges. This species is strongly associated with rangelands, as both in its native range (Europe) and within the area of range expansion (e.g., North America and New Zealand), *R. acris* can be found predominantly on dry and wet meadows and inhabits grazed and mown habitats^2,3^. This species is considered harmful to grazing animals wherever it is found. Specialized metabolites of this noxious weed (most notably ranunculin) are responsible for intoxications (resulting in intestinal disorders and respiratory failures) in cattle and horses^2^. Additionally, *R. acris* is resistant to commonly used herbicides. As a result, pastures and meadows infested by this weed are considered less valuable for agriculture because their usage is associated with substantial economic losses^2^.

Agricultural temperate meadows from Central Europe (from *Molinio-Arrhenatheretea* R.Tx. 1937 class), where *R. acris* can be predominantly found, grows on mesic and eutrophic soils^4^ that are naturally slightly saline^5^. As species existence on wetlands and meadows is mainly controlled by water resources, salinity and pH^6^, intensifying sodic saline stress (anthropogenic secondary salinity) can be treated as a major factor limiting plant growth, reproduction and the ability to withstand other stresses in these kinds of communities. In agricultural lands in temperate zone, the intensification of salinity (sodic salinity) is associated with numerous human activities^7^, mainly from saline water irrigation (if used^8^) and intensive winter maintenance of roads (from NaCl used predominantly for routine deicing in Europe and North America^9^). Consequently, pastures and meadows are affected by snowmelts flowing down roadsides and slopes of arable lands, causing considerable loads of Na^+^ and Cl^−^. Both ions are retained in groundwater and act as stressors for plants^8^. The intensity of salinity stress is modified by numerous environmental factors, i.e., precipitation, environmental conditions shaping evapotranspiration, groundwater relations and soil type^10^. Depending on these factors, sodic inputs can also trigger secondary effects that are associated with saline stress, i.e., a reduction in nutrient availability^8^. Unfortunately, the ability of many plant species from temperate pastures to withstand salinity stress is unknown.

Salt stress is associated with both the deterioration of water availability (physiological drought) and ion-specific toxicity^7,11^. The most outlined effects of salinity on plants can be seen for early ontogenetic stages, namely, germination and growth of seedlings^7^. Glycophytes are more susceptible to salinity at germination stage than halophytes^12,13^, but information on the ability of *R. acris* seeds to complete germination under salty environments is still lacking. During further developmental stages, the first noticeable sign of sodic saline stress in plants is wilting followed by chlorophyll loss, halted growth and leaf necrosis^7^. The abovementioned growth impairments result from multilayer disorganization of plant metabolism. A shortage of water sources reduces the availability and uptake of macro- and microelements^14^ and disrupts their transport and distribution within plants^7^. Sodic salinity hampers the maintenance of chlorophylls and negatively affects photosynthesis due to reduced water availability and Na^+^-dependent toxicity in chloroplasts^11^. Another well-documented sign of drought (including salinity-inducible signs) is morpho-anatomical changes in leaves^15^. Smaller and malfunctioning leaves cover less area and scavenge less sunlight; thus, fewer resources can be used for coping with environmental stresses. However, some *Ranunculus* species are able to fine-tune the architecture of their leaves to optimize their functioning under changing environments^15^.

When a given species is rapidly spreading within non-native range and/or is a nuisance weed (e.g., resistant to herbicides) and its occurrence is associated with economical losses (which is the case considering *R. acris*), it is very reasonable for good nature preservation and management to understand its behavior and the factors limiting its survival. General requirements for the growth of *R. acris* are known. This species has considerable tolerance to abiotic (e.g., varying edaphic conditions, cold and temporal drought) and biotic stresses (both pathogens and pests), allowing it to adjust well to different environments, such as meadows, pastures and other wet grassy habitats. However, it is still unclear whether this species is tolerant to saline habitats and sodic salinity. It is able to spread rapidly (even in habitats believed to be saline), but it can only be hypothesized whether it tolerates salinity. From a practical point of view, such information could be very valuable for monitoring programs and managing pastures (as recently suggested^16^), for which the quality of water sources plays a major role but is often omitted from an agronomical perspective. Thus, the aim of the present study was to elucidate the seed germination, growth and photosynthetic responses of *R. acris* to sodium chloride (NaCl). The following hypotheses were tested: 1) *R. acris,* as an invasive weed species, is able to withstand sodic salinity without any major growth limitations; 2) *R. acris* does not develop pathophysiological responses when subjected to sodic salinity. Testing these hypotheses was intended to answer the following questions: 1) Is *R. acris* a plant species tolerant to sodic-saline habitats? 2) Are there any functional adaptations pertaining to the primary functioning in this species that make it invulnerable to salinity?

## Results

### Ability of seeds to complete germination upon salinity

**Figure 1:**
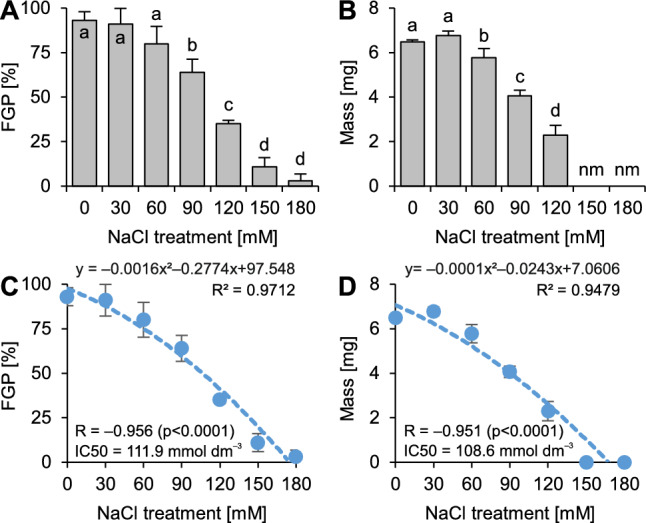
Effects of salinity (NaCl application) on germination and seedling weight of *Ranunculus acris* L. (**A**) Final germination percentage (FGP), (**B**) Fresh weight of seedlings, (**C**) Spearman rank correlation and IC_50_ value for FGP, (**D**) Spearman rank correlation and IC_50_ value for seedling growth. The correlation analyses and calculation of IC_50_ values were conducted on dataset merging measurements from all the studied variants (0–180 mmol dm^−3^ NaCl).

Seeds of *R. acris* completed germination well under 0–60 mmol dm^−3^ NaCl (Fig. 1). Greater doses of NaCl caused a significant reduction in the completion of germination, reducing it to near-zero values (Fig. 1). Very similar significant changes were observed for seedling FW, but detrimental effects of NaCl were observed even when plants were treated with solutions of 60 mmol dm^−3^ (Fig. 1), making seedling weight unmeasurable under 150 and 180 mmol dm^−3^ NaCl (the seeds completed germination but did not evacuate from the testa; Fig. 1).

FGP values and seedling weight correlated significantly (very strong negative correlation) with the concentration of the tested NaCl solutions (Fig. 1). The calculated IC_50_ values indicated the susceptibility of the studied species to salinity (Fig. 1).

### Plant growth and leaf morphology upon salinity

**Figure 2:**
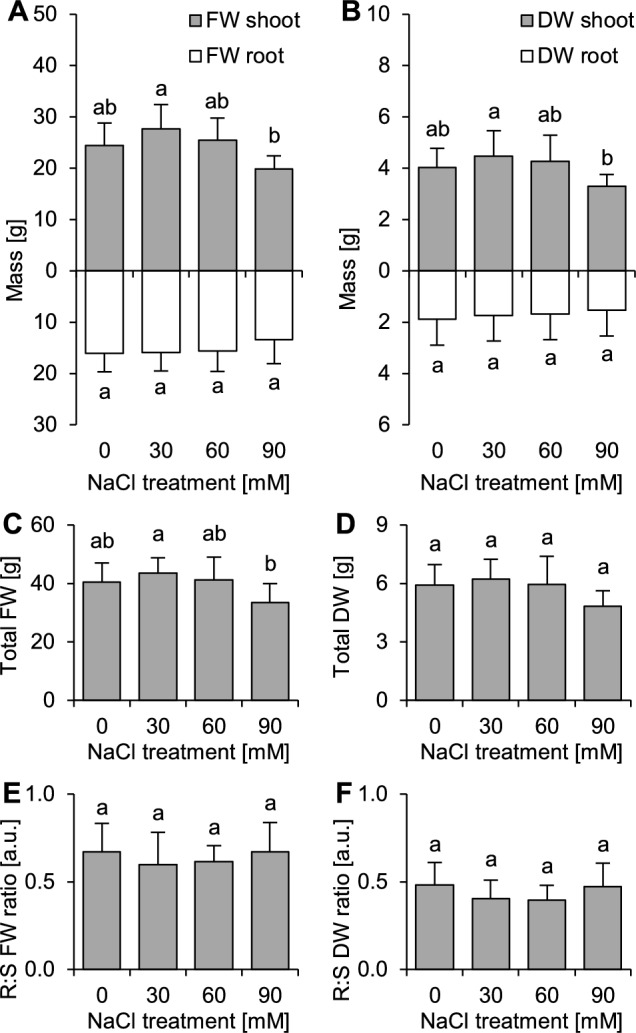
Effects of salinity (NaCl application) on the growth of *Ranunculus acris* L. (**A**) Fresh weights (FW) of roots and shoots, (**B**) Dry weights (DWs) of roots and shoots, (**C**) Total FW, (**D**) Total DW, (**E**) FW root:shoot ratio (FW R:S ratio), (**F**) DW root:shoot ratio (DW R:S ratio). The data are the means ± SDs (n = 8). Different letters indicate that the values are significantly different (*p* < 0.05; ANOVA with Tukey’s HSD post hoc test).

The plants showed the best performance when treated with 30 mmol dm^−3^ NaCl and the worst when treated with 90 mmol dm^−3^ NaCl (Fig. 2). Changes in FW but not DW values indicated alterations in tissue hydration upon salinity (Fig. 2). The allocation of resources (root:shoot FW and DW ratios) did not change with increasing NaCl application (Fig. 2).

**Figure 3:**
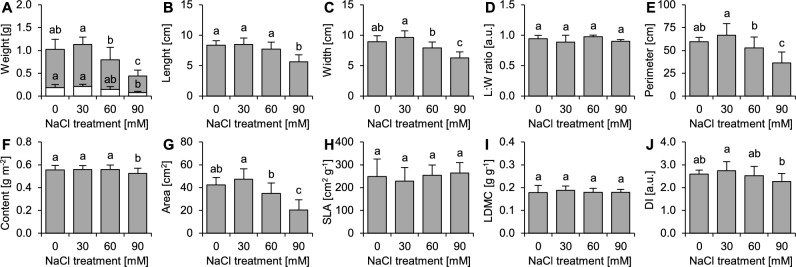
Effects of salinity (NaCl application) on leaf growth and morphometric and functional parameters of the leaves of *Ranunculus acris* L. (**A**) Fresh (FW) and dry (DW) weights (n = 8), (**B**) Length (n = 8), (**C**) Width (n = 8), (**D**) Length:width (L:W) ratio (n = 8), (**E**) Perimeter (n = 8), (**F**) Chlorophyll content (n = 24), (**G**) Area (n = 8), (**H**) Specific leaf area (SLA; n = 8), (**I**) Leaf dry matter content (LDMC; n = 8), (**J**) Dissection index (DI; n = 8). The data (**A**–**J** panels) are the means ± SDs. Different letters indicate that the values are significantly different (*p* < 0.05; ANOVA with Tukey’s HSD post hoc test).

The greatest growth of leaves (fresh weight, leaf width, perimeter and area values) was observed when the plants were treated with 30 mmol dm^−3^ NaCl, and the worst growth was observed when the plants were subjected to 90 mmol dm^−3^ NaCl (Fig. 3). The ratio between leaf length and width, specific leaf area (SLA) and leaf dry matter content (LDMC) were not significantly affected by the studied treatments (Fig. 3). A slight but significant decrease in chlorophyll content was observed in the plants treated with 90 mmol dm^−3^ NaCl (6% loss compared to the control; Fig. 3).

### Elemental composition and relations between mineral nutrition and growth

**Figure 4:**
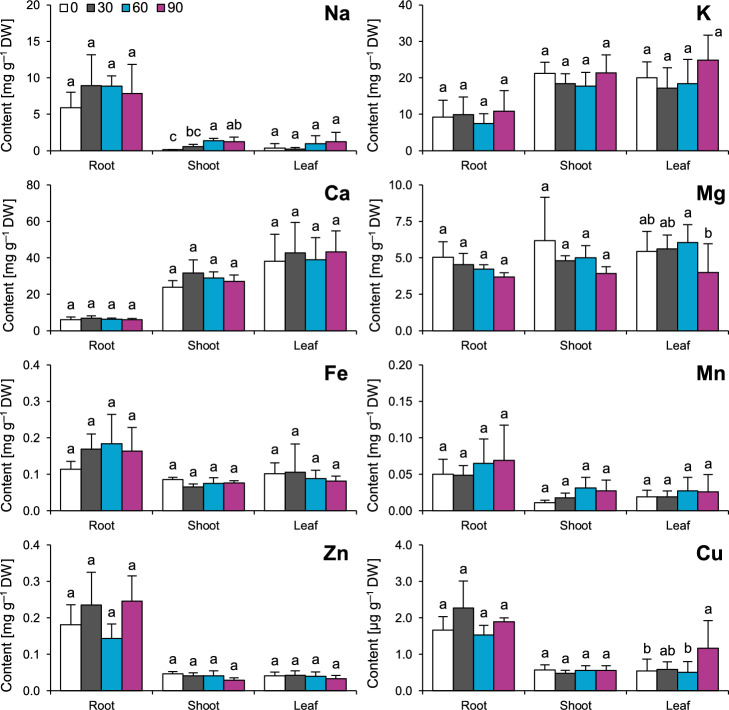
Effects of salinity (NaCl application) on the elemental composition of roots, shoots and leaves of *Ranunculus acris* L. The data are presented as the means ± SDs (n = 4 for roots and shoots; n = 8 for leaves). Different letters indicate that the values are significantly different (*p* < 0.05; ANOVA with Tukey’s HSD post hoc test).

NaCl-dependent changes in the contents of elements were sparingly significant. In shoots, a significant dose-dependent increase in Na content was observed, where 60 mmol dm^−3^ NaCl-treated plants contained significantly 884% more Na and 90 mmol dm^−3^ NaCl-treated plants contained significantly 775% more Na than the control plants (Fig. 4). In leaves, the only significant changes were observed for the contents of Cu and Mg, as the plants subjected to 90 mmol dm^−3^ NaCl contained 115% more Cu and 27% less Mg than the control plants (Fig. 4).

**Figure 5:**
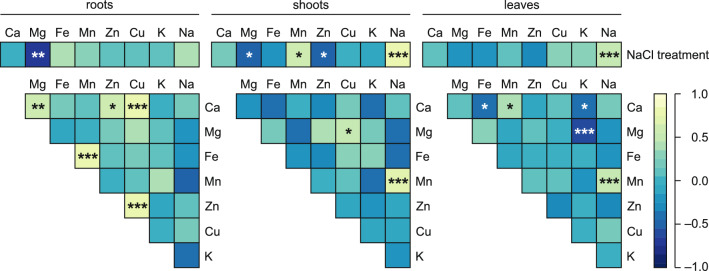
Correlations between NaCl and contents of elements and among contents of elements in the roots, shoots and leaves of *Ranunculus acris* L. subjected to the experimental NaCl gradient. The analyses were conducted on dataset merging measurements from all the studied variants (0–90 mmol dm^−3^ NaCl). The heatmap represents Pearson’s *R* values and significance (n = 16 for roots and shoots and n = 32 leaves). Correlations considered nonsignificant (*p* > 0.05) were not marked. * *p* < 0.05, ** *p* < 0.01, *** *p* < 0.005.

The contents of Mg correlated negatively with NaCl doses in roots (−0.691) and shoots (−0.509; Fig. 5), and the contents of Na correlated positively with Na doses in shoots (0.777) and leaves (0.521; Fig. 5). Additionally, significant correlations between Mn content (0.618) and Zn content (−0.546) and NaCl dose were observed in shoots (Fig. 5). Five significant correlations among elements were observed in roots, two in shoots and five in leaves (Fig. 5). Interestingly, significant positive correlations between Na and Mn in both shoots (0.683) and leaves (0.510) were observed (Fig. 5).

**Figure 6:**
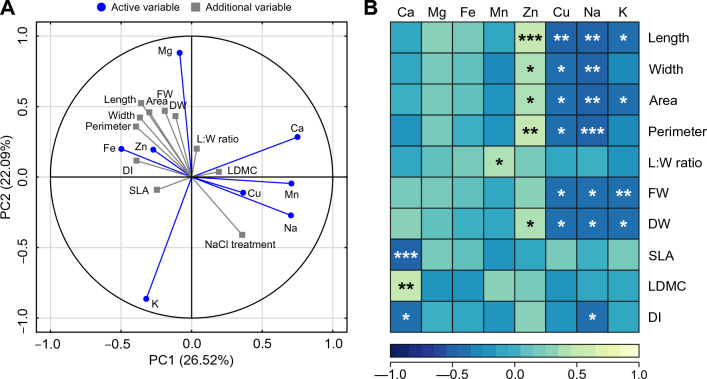
Correlation matrix of the content of the analyzed elements and leaf morphometrical traits plotted on two first principal components (PC) from principal component analysis (PCA; A) and Pearson correlations (**B**) between contents of elements in the leaves and morphometrical leaf traits of *Ranunculus acris* L. subjected to the experimental NaCl gradient. For PCA (**A**), each point represents a variable, and each vector represents a correlation between a given variable and PCs. Color-marked traits were used for the generation of PCs (active variables), and gray-marked traits were plotted as additional variables. Percentages presented in parentheses denote the explained variance. For details pertaining to the loadings of PC1, PC2, PC3 and PC4, see Supplemental Table 1. The correlation analyses (**B**) were conducted on dataset merging measurements from all the studied variants (0–90 mmol dm^−3^ NaCl). The heatmap represents Pearson’s *r* values and significance (n = 32). Correlations considered nonsignificant (*p* > 0.05) were not marked. * *p* < 0.05, ** *p* < 0.01, *** *p* < 0.005.

The first two components of PCA explained 48.61% of the total variance in the elemental composition of the leaves (Fig. 6). The most important loading for PC1 was Ca, followed by Mn and Na, and for PC2, the most important loadings were Mg and K (Supplemental Table 1). PC3 explained 16.77% of the variance, and PC4 explained 12.75% of the variance; all four PCs explained 78.13% of the variance (Supplemental Table 1). The contents of Na, Mn and Cu coordinated well with NaCl treatment and were positively correlated with PC1 and negatively correlated with PC2 (Fig. 6). The opposite was observed for the contents of Fe, Zn and Mg as well as leaf length, leaf width, area, FW, DW, perimeter and dissection index (Fig. 6). SLA and the content of K correlated negatively with both PCs, and the opposite was observed for the content of Ca, leaf length:width ratio and LDMC (Fig. 6).

The content of Ca correlated negatively with SLA (−0.528) and DI (−0.361) but positively with LDMC (0.453; Fig. 6). The content of Mn correlated only with the leaf length:width ratio (0.394; Fig. 6). The content of Zn correlated positively with the length (0.486), width (0.398), area (0.419), perimeter (0.459) and DW of leaves (0.361; Fig. 6). The opposite was observed for Cu, Na and K, which correlated negatively with the length (−0.463 for Cu, −0.464 for Na, −0.443 for K), width (−0.416 for Cu, −0.462 for Na), area (−0.390 for Cu, −0.451 for Na, −0.417 for K), perimeter (−0.370 for Cu, −0.490 for Na), FW (−0.393 for Cu, −0.412 for Na, −0.449 for K) and DW of leaves (−0.376 for Cu, −0.397 for Na, −0.424 for K; Fig. 6).

### Fluorescence of chlorophyll *a* upon salinity

**Figure 7:**
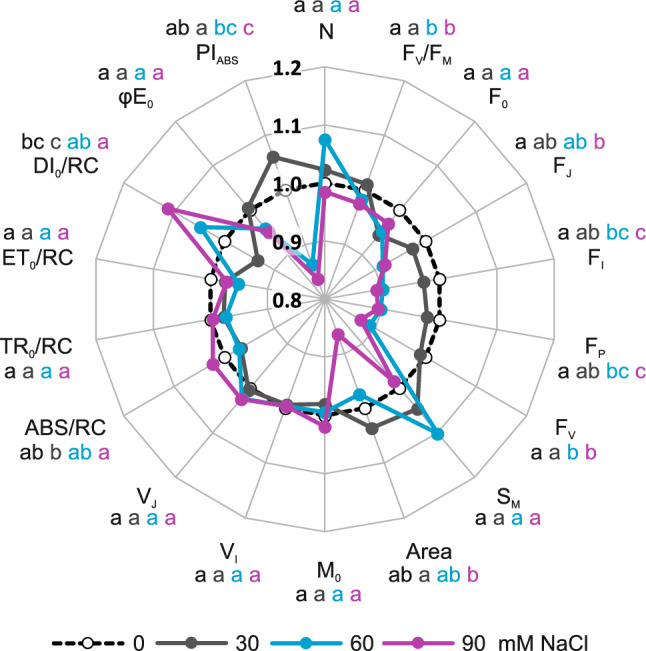
Effects of salinity (NaCl application) on chlorophyll *a* fluorescence parameters measured in the leaves of *Ranunculus acris* L. using the OJIP method. Relative values (means) were calculated as ratios between mean values measured on plants subjected to NaCl treatments and mean values measured on plants not treated with NaCl (control). Values with different letters are significantly different between the treatments within a given species at *p* < 0.05 (n = 16; ANOVA with Tukey’s HSD post hoc test). The earlier letter indicates a significantly higher value of the parameter. The color of letter-based statistical indicators refers to the respective experimental variant as indicated in the legend.

Dose-dependent significant changes were observed for F_J_, F_I_ and F_P_, where the greater the dose of NaCl was, the lower the values of those parameters were (Fig. 7). For F_V_/F_M_ and F_V_, significantly negative changes were observed when the plants were subjected to 60 and 90 mmol dm^−3^ NaCl. Changes reflecting the best shoot and leaf growth were observed for Area, ABS/RC, DI_0_/RC and PI_ABS_, where 30 mmol dm^−3^ NaCl-treated plants performed the best, followed by those treated with 0, 60 and 90 mmol dm^−3^ NaCl (Fig. 7).

### Shift in CSR strategy upon salinity

**Figure 8:**
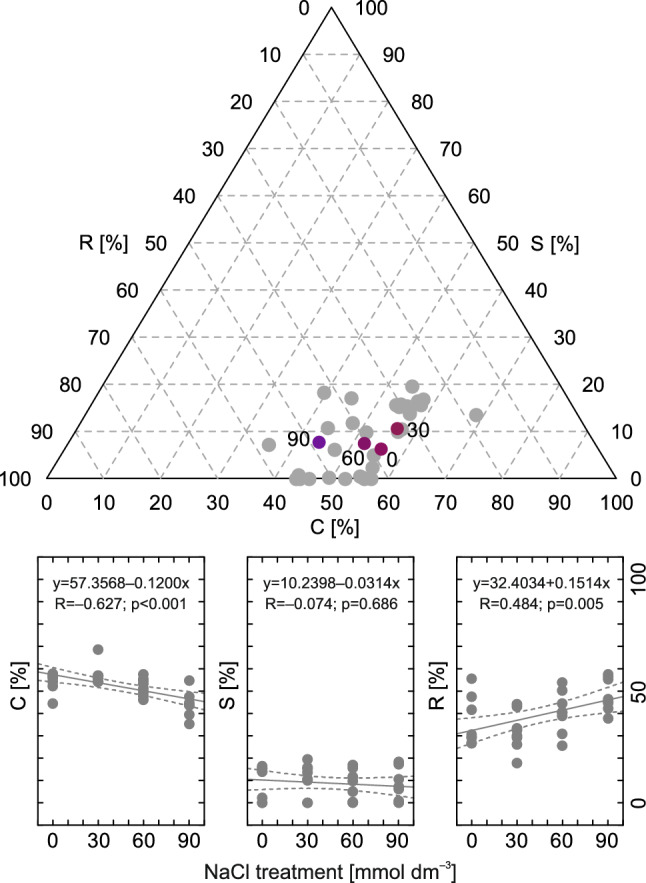
Ternary diagram of C-, S- and R-selection for *Ranunculus acris* L. subjected to salinity (NaCl application) and Spearman rank correlation between C, S and R selection and salinity. Each point in the ternary diagram represents the relative proportion (%) between the C, S and R scores calculated for single individuals from all experimental variants using raw measurements (32 in total; gray dots) and for means characteristic of each experimental variant (0, 30, 60 and 90 mmol dm^−3^ NaCl; colored dots).

The control plants showed a CR-type strategy (55.60% C, 6.20% S, 38.10% R; Fig. 8). A slight shift toward a more balanced strategy (C/CSR) was observed for the plants treated with 30 mmol dm^−3^ NaCl (56.30% C, 10.50% S, 33.20% R; Fig. 8). Greater NaCl doses caused a gradual reduction in competitiveness and an increase in ruderal behavior (52.10% C, 7.40% S, 40.50% R in plants treated with 60 mmol dm^−3^ NaCl and 44.00% C, 7.70% S, 40.30% R in plants treated with 90 mmol dm^−3^ NaCl; Fig. 8). The changes in competitiveness (C) were negatively correlated with NaCl dose (−0.627), while the opposite was observed for ruderal behavior (R; 0.484; Fig. 8).

## Discussion

Overall, it seems that *R. acris* is able to cope with slight sodic salinity (or even take advantage of subsaline soils), but it is not a salinity-tolerant species or typical halophilic plant (contrary to hypothesis 1, this species encounters NaCl-dependent limitations). Although past studies have reported that *R. acris* prefers patches where the soil salt concentration is not marked^17^, this is the first report showing that the absence of this species in saline patches is in fact caused by increasing salinity.

*R. acris* presented a low ability to complete germination when compared to halophilic plants^12^, but its completion of germination upon salinity was still better than that of many wild-living (glycophytic) plants from similar habitats, including *Potentilla reptans* L.^18^, *Plantago lanceolata* L.^19^ and *Trifolium repens* L.^20^. Progressing salinity not only negatively affected the completion of germination but also decelerated the growth of seedlings in this species (contrary to hypothesis 1). As the IC_50_ values ranged ca. 110 mmol dm^−3^ NaCl for both the ability of seeds to complete germination and the subsequent growth of seedlings, it can be predicted that worsening of soil water resources is likely to reduce the population size of this species due to inhibition of seed germination. On the other hand, in nondisturbed (naturally subsaline) meadows, its tolerance to sodic salinity allows successful establishment.

Similar conclusions can be drawn for further vegetative growth. The studied species seems to have a niche similar to several other commonly occurring dicotyledonous plants that are nonspecialized general meadow species. However, taking into account the results of the present study (contrary to hypothesis 1), it can be stated that *R. acris* is probably more susceptible to saline conditions than *Trifolium repens* or some *Taraxacum* and *Hieracium* species and represents salinity tolerance similar to *Achillea millefolium* L.^21^ or *Trifolium pratense* L. (in which growth inhibition due to 20 mmol dm^−3^ NaCl treatment was observed^22,23^). In the field, observations of *R. acris* on roadsides and road ditches (where anthropogenic salinity from road maintenance is a serious problem) are very common in Central Europe^17,24,25^. Such habitats are often characterized by higher salinity (which is intuitively contrary to described growth relations), but they are also characterized by higher water ability, which can help *R. acris* survive and reproduce^26^. Notably, the growth of this species was substantially inhibited when the plants were grown on 90 mmol dm^−3^ NaCl-treated soil. This clearly indicates that the studied species is not halophilic, as inhibition of plant growth is characteristic of nonhalophytes^27,28^. This can be the result of Na toxicity itself^29^ or Na-inducible physiological drought caused by extraxylary (reversible) losses of hydraulic conductivity^30^, which is very likely considering changes in shoot FW and DW values. Under field conditions, such growth reduction can contribute to a reduction in plant fitness and the ability to compete with other species but not necessarily to reproduce^31^. It must be noted that *R. acris* was still able to survive under the highest tested salinity dose. Most likely, this was due to its ability to maintain the functioning of cells and physiological avoidance of desiccation-induced damage^30^. In summary, it seems that *R. acris* is able to form persistent populations in temporally slightly and moderately saline habitats, including those polluted by human activity. As retardation of plant growth can be associated with a reduced ability to form stable populations and spread in the studied species^32^ and the anatomical properties of shoots and stems of *R. acris* were shown to be a crucial adaptation to win competition in an established meadow environment (where supporting tall structures play a key role^33^), the observed inhibition of growth under the highest studied salinity suggests that *R. acris* is likely to encounter greater pressure from direct competition with increasing salinity. In this context, pastures in which water management practices are poor will not serve adequate habitat for this species.

Surprisingly, we found that the growth of *R. acris* was stimulated by the application of 30 mmol dm^−3^ NaCl (an effect that was not hypothesized during the design of the study). Although such stimulating effects caused by low doses of NaCl (and some other salts) are known phenomena and have also been observed in some other wild and cultivated plant species, including glycophytes^34^, finding a satisfactory explanation for the growth stimulation of plants under saline or subsaline conditions is a difficult task, even in halophytes^35^. It must be noted that these effects are species-specific and refer to a relatively narrow concentration window of selected salts, including NaCl (20–50 mmol dm^−3^^34^). Considering the presented results and taking into consideration the very low Na contents of the soil we used, it can be presumed that *R. acris* is a species whose optimal niche is realized in subsaline soils (where the salt load is less than the threshold for soil salinity; ca. 36 mmol dm^−3^ NaCl^36^), similar to typical soils of temperate meadows, pastures and rangelands. This means that *R. acris* is very well adapted to habitats that are not severely disturbed by anthropogenic salt loadings.

On the basis of this study, it can also be stated that *R. acris* does not experience any serious problems with mineral nutrition that could be associated with progressing sodic salinity (which is in line with hypothesis 2). This is rather unusual, as sodic salinity halts adequate acquisition of both macro- and micronutrients^37^. However, several (but not all) halophytic or halotolerant plants are known to maintain their mineral nutrition rather stably under sodic salinity, e.g., *Tamarix gallica* L.^38^, *Sesuvium portulacastrum* (L.) L.^39^ or *Crithmum maritimum* L.^40^. In this context, the physiology of *R. acris* resembles the functioning of halophytes. This can be very valuable for this species, as balanced mineral nutrition is known to ensure the survival of individuals and their reproduction^41^. The only observable effects of increasing salinity were the accumulation of Na and Cu in the aerial parts of the studied individuals (but not always in the youngest leaves, as the variation in Na content was greater than that in whole shoots). Similar results were observed for other mesic glycophilic plant species, e.g., *Solanum lycopersicum* L. or *Medicago sativa* L.^42,43^. This suggests that toxic amounts of Na are likely to accumulate in well-developed, older leaves more than in younger leaves^44^. Thus, older leaves are more likely to senesce due to Na intoxication of mesophyll cells^44^, which is counterbalanced by the generation of younger leaves not exposed to Na toxicity, which is in line with the results of the present study. It must be noted that most Na ions did not enter long-distance transport on the root‒shoot axis and remained in roots (the content of Na in roots was very stable), which can be seen as some kind of adaptation conferring avoidance of intoxication of aerial organs^46^. However, increasing salinity triggered the gradual accumulation of Na in shoots. As increasing salinity stress deteriorated the functioning of the photosynthetic apparatus, it can be proposed that storage of excessive Na in vacuoles (adaptive mechanism of some halophytes^45^) is probably not efficient in *R. acris*. The most likely mechanism responsible for the transportation of Na to aerial organs is uncontrolled entrance of ions via bypass flow in Casparian strips of endodermis^47^ followed by compulsive xylem loading. This is the most likely explanation for why the accumulation of Na in roots failed to be sufficient and thus to guarantee proper physiological functioning when this species faces progressing salinity. The observation of progressive Na accumulation accompanied by a lack of serious nutritional disorders strongly suggests that the following functional limitations are probably effects of Na ions themselves (or they are only slightly modified by other assayed nutrients).

In general, many *Ranunculus* species are known to be highly plastic—the architecture of their leaves is fine-tuned in response to environmental conditions, e.g., water availability or salinity. Changes in leaf morphology, including the dissection of leaves, have already been described for many *Ranunculus* species^15,48–51^. In all those species, the greater the availability of water in soil, the greater the dissection of the leaves, and vice versa. Our study showed that leaf dissection was affected by changing NaCl application, but these changes were only slightly pronounced compared to those in nature^15^. A similar coincidence between progressing salinity and leaf size was observed in other plant species from temperate meadows^22^ as well as in other *Ranunculus* species^31^. Nevertheless, dissection of leaves seems to be associated with progressing Na intoxication of plants, as DI was negatively correlated with the content of Na in the leaves. Although leaf shape and size were suggested to be adaptations contributing to salinity tolerance due to a reduction in evaporation-dependent water stress^31^, the observed changes in DI values cannot be seen as an adjustment contributing positively to plant fitness under salinity due to the small size of effects and other associated effects (reduced area of leaves and worsened functioning of photosynthetic apparatus; contrary to hypothesis 2).

Our results demonstrated that leaves from plants subjected to saline stress were not only smaller but also developed dysfunctions at the level of the photosynthetic apparatus (contrary to hypothesis 2). Several previous works suggested that apart from F_V_/F_M_, PI_ABS_ and DI_0_/RC can be used as proxies of the status of the photosynthetic apparatus under salinity stress^52^. The presented data pertaining to chlorophyll *a* fluorescence indicated that *R. acris* subjected to 60 and 90 mmol dm^−3^ NaCl developed a pathophysiological stance. The same coinciding increase in DI_0_/RC and decrease in PI_ABS_ values were observed in other NaCl-stressed plant species^52,53^. Correlation analysis strongly suggests that Na is a major source of such a mode of action. Thus, it indicates osmotic- and/or nutritional-caused limitations in plant cells, leading to the inhibition of electron transport, decreased performance and increased energy dissipation^52^. Additionally, as a positive correlation between Mn and Na was observed in shoots and leaves, it can be proposed that the negative effects of Na are additionally modified by Mn. The abovementioned limitations are other arguments for treating *R. acris* as a species that is not well adapted to salinity stress. Interestingly, malfunction in this species was not associated with phenotypically distinguishable changes, including a lack of serious changes in chlorophyll content (except 90 mmol dm^−3^ NaCl). In contrast to the evident deterioration of plant functioning under high salt doses, plants subjected to 30 mmol dm^−3^ NaCl encountered slight stimulation of the selected parameters (not hypothesized to occur during the design of the study). Similar stimulation (e.g., when considering F_V_/F_M_ values) was observed in *Beta vulgaris* L., which is a plant species that prefers mild salinity^54^. This is another argument for seeing *R. acris* as a species that is able to take advantage of subsaline conditions, including temperate meadows and pastures.

Generally, determination of the CSR strategy is considered useful for comparison of different plant species^55^; however, it can also be used for monitoring the response to environmental stressors, including salinity^56^. In the case of *R. acris*, intensifying salinity (60 and 90 mmol dm^−3^ NaCl) caused a slight but significant shift from a competition-oriented to a disturbance-oriented strategy. Rephrasing it, salinity decreased investments in the development of large leaves and growth in general, promoting the development of smaller leaves, whose loss due to progressing Na intoxication would pose a lesser challenge to plant homeostasis than the loss of larger leaves. This can be associated with the lesser efficiency of the photosynthetic apparatus (confirmed in this study), shortage of photosynthesis products and intensification of needs associated with processes other than growth. It is also worth noting that the plants did not develop any adjustment associated with stress tolerance. The opposite was observed in plants subjected to 30 mmol dm^−3^ NaCl, which promoted greater competition and balanced its development toward the C/CSR-type strategy. This means that under subsaline conditions (e.g., in meadows), this species is in a greater competitive stance than when it grows in nonsaline or saline habitats. This probably helps it monopolize access to resources (mostly space and light) in a wide spectrum of natural and man-made habitats.

Looking for the practical context of this study, it is worth noting that some other recent studies showed that *R. acris* can be very suitable for monitoring Na availability in soils; the greater the availability of Na in soil, the greater the leaf concentration of Na^16^. Our study agrees with those findings but also suggests that spatial inconsistency of plant occurrence (due to inhibition of seedling establishment) or easily catchable conspicuous differences in plant physiognomy of *R. acris*, such as plant size and leaf morphology (due to Na intoxication), can be a signal suggesting progressing salinization of habitat.

## Conclusions

The present study shows that the growth, development and functioning of *R. acris* are seriously deteriorated upon sodic salinity, but this species can withstand subsaline and (constantly or temporarily) slightly saline habitats. However, all malfunctions observed in *R. acris* seem to be effects of simple Na intoxication but not effects of more complicated collapse of mineral nutrition. Finally, it leads to a reduction in its competitive ability and switches to a ruderal (but not stress-tolerant) strategy until tolerance limits are reached. All this is associated with measurable, dose-dependent decreases in leaf size and functioning. In conclusion, it can be suggested that the lack of this species in suitable habitats does not necessarily mean that a given habitat does not suffer from stress; in fact, the opposite can take place. All this means that observations of *R. acris* in the field can provide indirect but valuable information about the processes taking place in nature, especially when gradual pollution (e.g., accumulation of Na due to fertilization and management of roads) and transformation of the landscape (inadequate drainage, transformation of terrain relief, fragmentation of habitats due to the increasing density of road networks) takes place. Ultimately, there is a lack of studies on stress response-associated biochemical events upon salinity exposure in meadow plants, including *R. acris*, thus the mechanisms underlying the trade-offs between growth and physiological regulation of the stress response in this species and plants from its habitat remain unclear. Therefore, further research should be carried out to determine how stress-coping mechanisms (e.g., primary and specialized metabolism and antioxidant system) are involved in plant reactions to salinity and the implications of such responses.

## Materials and methods

### Seed material

Seeds of *R. acris* (mean seed weight ± SD = 1.18 ± 0.21 mg) were collected from a seminatural nonsaline habitat located in the Wilczków peat bog area (51°56’N, 18°52’E) in central Poland in a privately owned nonprotected area (permit for collection of seeds was not required). This species is not endangered or at risk of extinction or protected. The study complies with relevant institutional, national, and international guidelines and legislation. The voucher specimen of the studied species was deposited in Herbarium Universitatis Lodziensis, LOD, with accession number 158114 (collected and identified by M. Wala). To fulfil the requirement of representative sampling, mature seeds were collected from at least 150 individuals of one single and large population. Then, the seeds were stored in the laboratory (23 °C; relative humidity of 40–60%) for 14 days and, then, stratified in a refrigerator (4 °C) for 120 days. The seeds were mixed before the tests to fulfil the randomization requirement.

### Investigation of the ability to complete germination under sodic salinity

The ability of seeds to complete germination under increasing salinity was tested to estimate the capability of the studied species to colonize saline habitats. The experiment was conducted using the standard Petri dish method. For each experimental variant, experiments were repeated four times, and each repetition consisted of one Petri dish. The seeds were sown on Petri dishes (ø = 5 cm; 25 seeds per dish) lined with two layers of filter paper (Whatman no. 1; GE Healthcare, Chicago, IL, United States). Then, 1.5 cm^3^ of the tested solutions were introduced. The effects of salinity were studied using a series of NaCl solutions (prepared using analytical grade NaCl and low conductivity water; < 0.08 µS cm^−1^) ranging 0–180 mmol dm^−3^ with an interval of 30 mmol dm^−3^. The dishes were sealed with parafilm and placed in a germination cabinet under a fully controlled thermophotoperiod (16 h light phase at 25 °C and 8 h dark phase at 15 °C; light was supplied with white fluorescent lamps, PAR = 40 μmol m^−2^ s^−1^, measured with FluorPen; PSI, Drasov, Czech Republic^57^). Completion of germination was counted at the end of the experiment (21 days after seed sowing). The seeds were checked for any signs of their nonviability (seed softness and brownish embryo color); however, no such situation was observed. The final germination percentage (FGP) was calculated, where 0 and 100 indicate minimal and maximal completion of germination, respectively. The half maximal inhibitory concentration (IC_50_) was calculated as 50% inhibition of the control variant using the quadratic equation of the polymonial trendline (as it yielded a greater R^2^ value than the linear model).

### Establishment of seedlings in pot culture and experimental setup

To test the effects of increasing salinity on morphophysiological attributes (and thus tolerance of the studied species to saline habitats), a pot experiment was conducted. The experiment was conducted under fully controlled photothermal conditions. The light (16/8 h light–dark cycle) was supplied with LED lights (Growy; Neonica, Łódź, Poland) with PAR of 100 mmol m^−2^ s^−1^ (measured with FluorPen FP100; Photon Systems Instruments, Drásov, the Czech Republic). The seeds were sown on nonsaline commercially available garden soil (62.8 mg Na kg^−1^ soil dry weight). The seedlings were established in standard 103-well garden trays (well capacity of 20 cm^3^); three seeds were sown per well (309 in total), and after emergence (emergence of seedlings was high, ca. 75% of sown seeds), the number of seedlings was manually reduced to one per well using tweezers. Then, the 7-day-old seedlings were transplanted to 1.5 dm^3^ plastic pots (one plant per pot).

The 42-day-old seedlings were randomly divided into four groups and subjected to experimental treatments. In total, 32 plants were used for experimentation; the experiment consisted of four replications, and each replication consisted of two plants per experimental variant (n = 8 per experimental variant). The youngest leaf (at a very early stage of development) from each individual was marked with a loose polypropylene band (these leaves were used for determination of chlorophyll content, measurements of chlorophyll *a* fluorescence and destructive analyses). The plants were treated with NaCl solutions of 0, 30, 60 and 90 mmol dm^−3^ prepared using tap water used for plant watering and analytical grade NaCl. The range of treatments was selected on the basis of the abovementioned germination experiment, taking only concentrations lower than the IC_50_ values for the germination and growth of seedlings. The plants were treated with NaCl solutions (50 cm^3^) at 6-day intervals (8 doses in total). Six days after the last treatment, 96-day-old seedlings were subjected to the analyses.

The total number of plant individuals studied in this work was 32 (eight per experimental variant in total representing eight independent replications).

### Measurements of growth and leaf morphometric traits

Growth and morphometric analyses were conducted to estimate the productivity of the studied species to check if there were any adjustments in its architecture that could improve tolerance to increasing salinity. For morphometrical analysis, the leaves marked with polypropylene bands (fully expanded on the 96th day of the experiment) were excised (leaf blades without petioles). Then, they were weighed on a precise balance to measure their fresh weight (FW). Subsequently, the leaves were scanned with a CI-202 portable laser area meter (CID Bio-Science, Camas, WA, USA) to record their length, width, length to width ratio, area and perimeter. Then, each leaf was dried separately at 70 °C until the dry weight (DW) was recorded. Leaf dry matter content (LDMC) and specific leaf area (SLA) were calculated according to standard methods^58^ and expressed in mg DW g^−1^ FW (LDMC) and cm^2^ mg^−1^ DW (SLA). The dissection index was calculated according to the formula from the literature^59^; the higher the value of DI is, the greater the dissection of the leaf blade, where a value of 1 indicates an ideally circular shape. For all analyses of leaf traits, eight leaves (each from a different plant) per treatment were used.

The plants were extracted from pots, gently washed out and blotted dry with paper towels. Then, the roots were precisely excised using a razor blade. The roots and shoots were weighed to obtain FW and dried using the procedure used for the leaves. Shoot FW and DW were calculated as the sum of excised leaf and remaining plant material. Total shoot and root FW and DW were calculated as the sum of roots and shoots. The shoot:root (S:R) ratios were calculated using both FW and DW values. For all analyses of plant growth traits, eight plants per treatment were used.

### Calculation of CSR strategy

To estimate any change in the overall adjustment to the tested conditions, changes in life strategy referring to competitor-stress-tolerator-ruderal (CSR) theory (sensu Grime, 1974^60^) were evaluated. CSR scores were calculated using the ‘StrateFy’ calculator tool^55^ using values of leaf area, LDMC and SLA. CSR scores were calculated for each leaf sampled in this study (eight per experimental variant) as well as for mean values of these traits in a given experimental variant (0, 30, 60, 90 mmol dm^−3^). The estimated type of strategy was calculated using the equations of the ‘StrateFy’ tool, and the CSR scores were then plotted on a ternary diagram. The higher the C, S and R values are, the more a given variant is biased toward competitor, stress-tolerant or ruderal strategies, respectively.

### Measurements of chlorophyll content and analysis of chlorophyll *a* fluorescence

To investigate primary physiological stress upon increasing salinity, analyses associated with the structure and functioning of the photosynthetic apparatus were conducted. Analyses of chlorophyll content and chlorophyll *a* fluorescence were conducted prior to destructive sampling. The chlorophyll content was measured with a CCM-300 portable chlorophyll content meter (Opti-Sciences Inc., Hudson, NH, USA). The values were expressed in mg m^−2^. The chlorophyll content was measured on three interveinal spots of one fully developed leaf per plant and eight plants per treatment. The changes in the polyphasic rise in chlorophyll *a* fluorescence (OJIP) were inspected with a handheld PAM-type fluorometer (FluorPen FP100; Photon System Instruments; Drásov, Czech Republic). The leaves were dark-adapted for 20 min prior to analysis with standard detachable clips (appropriate for this purpose). The measurements (see Supplemental Table 2 for description of the studied parameters) were recorded using the preprogrammed protocol of the device (according to the literature^61^). The OJIP parameters (Supplemental Table 2) were measured on two interveinal spots of one fully developed leaf per plant and eight plants per treatment. The fluorescence fingerprint of the measured parameters was presented as the means of values normalized to the values measured on the control plants (grown without NaCl addition) using a radar plot^62^.

### Analysis of elemental composition

Analysis of the elemental composition was conducted to determine the reason for the observed growth and morphological and functional impairments. The content of elements in the roots, shoots and leaves (from morphometrical leaf blade analyses) was determined as previously reported^63^. The samples were wet-digested at 140 °C in a HNO_3_ and HClO_4_ mixture (4:1 ratio, v/v). Concentrations of calcium (Ca), magnesium (Mg), iron (Fe), manganese (Mn), zinc (Zn), copper (Cu), sodium (Na) and potassium (K) in the mineralizates were quantified with an air/acetylene flame-equipped atomic absorption spectrometer (SpectrAA 300; Varian Australia Pty. Ldt. ; Mulgrave, VIC, Australia). A deuterium lamp was used for background correction. The content of each element was expressed in mg g^−1^ DW µg g^−1^ DW. The measurements were performed on four samples of the roots and the shoots (where each sample was a bulk sample of two plants in equal proportions) and eight samples of the leaves (each from a separate plant).

### Statistical analysis

Each experimental variant consisted of eight real biological replicates (plants per treatment; n = 8). The normality of the data was examined with Kolmogorov–Smirnov’s test, and the homogeneity of variances was examined with Brown–Forsythe’s test. The differences between treatments were detected by one-way ANOVA followed by Tukey’s HSD post hoc test. Differences between variants were accepted as significant at *p* < 0.05, and all differences described as significant pertain to this value. To study the correlation among the contents of elements, the Pearson correlation was calculated. To study the correlation between elements and applied NaCl treatments, the Spearman sum rank correlation was calculated. Correlations were accepted as significant at *p* < 0.05, *p* < 0.01 and *p* < 0.005, and all correlations described as significant pertain to those values. To determine relations between morphometrical leaf traits and elemental composition, Pearson correlation was calculated (with the same criteria for significance as for Spearman testing), and the Principal Component Analysis (PCA) was conducted using correlation matrices. The dataset from elemental composition was used for the generation of principal components (explaining variables), and the dataset from morphometrical analysis was used as a source of additional (explained) variables. All statistical analyses were conducted using Statistica™ v. 13.3 (Tibco Software Inc., Palo Alto, CA, USA).

### Supplementary Information

Supplementary Information 1.

Supplementary Information 2.

## Data Availability

The datasets used and analyzed during the current study is available from the corresponding author on reasonable request.
