# Supplementary material for: Experimental investigation of the responses of meadow buttercup (Ranunculus acris L.) to sodic salinity and its implications for habitat monitoring
Source: Sci Rep. 2023 Sep 20;13:15611. doi: 10.1038/s41598-023-42738-2 (PMC10511526; doi:10.1038/s41598-023-42738-2)
Supplement: Supplementary file 1 — Supplementary Information 1. [file 41598_2023_42738_MOESM1_ESM.docx]

**Supplemental Table 1. Loading values for the four first components (PC1, PC2, PC3 and PC4) of Principal Component Analysis (PCA) conducted for elemental composition of leaves.**

| Principal component | Explained variance | Ca | Mg | Fe | Mn | Zn | Cu | Na | K |
| --- | --- | --- | --- | --- | --- | --- | --- | --- | --- |
| PC1 | 26.52% | 0.266 | 0.003 | 0.117 | 0.236 | 0.035 | 0.063 | 0.233 | 0.048 |
| PC2 | 22.09% | 0.046 | 0.439 | 0.023 | 0.001 | 0.021 | 0.007 | 0.042 | 0.421 |
| PC3 | 16.77% | 0.020 | 0.039 | 0.088 | 0.075 | 0.435 | 0.334 | 0.009 | <0.001 |
| PC4 | 12.75% | 0.090 | 0.005 | 0.421 | 0.263 | 0.083 | 0.007 | 0.130 | 0.002 |
