# Supplementary material for: Experimental investigation of the responses of meadow buttercup (Ranunculus acris L.) to sodic salinity and its implications for habitat monitoring
Source: Sci Rep. 2023 Sep 20;13:15611. doi: 10.1038/s41598-023-42738-2 (PMC10511526; doi:10.1038/s41598-023-42738-2)
Supplement: Supplementary file 2 — Supplementary Information 2. [file 41598_2023_42738_MOESM2_ESM.docx]

**Supplemental Table 2. Parameters derived from the OJIP transient used in this study, formulas of their calculation and definitions.**

| OJIP parameter | Formula | Definition |
| --- | --- | --- |
| F_0_ (=F_O_) | F_0_=F at 50μs (=F at O-step) | Fluorescence intensity at O-step (at 50 μs) (=Minimal fluorescence intensity) |
| F_J_ | F_J_=F at 2ms (=F at J-step) | Fluorescence intensity at J-step (at 2 ms) |
| F_I_ | F_I_=F at 60ms (=F at I-step) | Fluorescence intensity at I-step (at 60 ms) |
| F_M_ (=F_P_) | F_M_=F at 1s (=F at P-step) | Fluorescence intensity at P-step (at 1000 μs) (=Maximal fluorescence intensity) |
| F_V_ | F_V_=F_M_–F_0_ | Maximal variable fluorescence |
| V_J_ | V_J_=(F_J_–F_0_)/(F_M_–F_0_) | Relative variable fluorescence at J‑step (2 ms) |
| V_I_ | V_I_=(F_I_–F_0_)/(F_M_–F_0_) | Relative variable fluorescence at I‑step (60 ms) |
| F_V_/F_M_ | - | Maximum quantum yield of primary PSII photochemistry |
| M_0_ | M_0_=TR_0_/RC–ET_0_/RC | Approximated initial slope of the fluorescent transient |
| Area | - | Area between fluorescence curve and F_M_ (background subtracted) |
| S_M_ | S_M_=Area/(F_M_ - F_0_) | Standardized area above the fluorescence curve between F_0_ and F_M_ |
| N | N=S_M_*M_0_*(1/V_J_) | Number of Q_A_ redox turnovers until F_M_ is  reached |
| ϕ_E0_ | ϕ_E0_=[1–(F_0_/F_M_)]*ψ_0_ | Quantum yield for electron transport from Q_A_ to plastoquinone at t = 0 |
| PI_ABS_ | PI_ABS_=γRC/(1−γRC)*ϕ_P0_/(1−ϕ_P0_)*ψ_0_/(1−ψ_0_) | Performance index of electron flux from PSII based to intersystem acceptors |
| ABS/RC | ABS/RC=M_0_*(1/V_J_ )*(1/ϕ_P0_ ) | Photon flux absorbed by PSII antenna chlorophyll per RC at t = 0 |
| TR_0_/RC | TR_0_/RC=M_0_*(1/V_J_) | Trapping flux leading to Q_A_ reduction per RC at t = 0 |
| ET_0_/RC | ET_0_/RC=M_0_*(1/V_J_ )*ψ_0_ | Electron transport flux per RC at t = 0 |
| DI_0_/RC | DI_0_/RC=(ABS/RC)–(TR_0_/RC) | Dissipated energy flux per RC at t = 0 |
